# 3D Construction Printing Standing for Sustainability and Circularity: Material-Level Opportunities

**DOI:** 10.3390/ma16062458

**Published:** 2023-03-20

**Authors:** Mariana Fonseca, Ana Mafalda Matos

**Affiliations:** 1 Associação CECOLAB, Collaborative Laboratory Towards Circular Economy, 3405-155 Oliveira do Hospital, Portugal; 2 CONSTRUCT-Labest, Faculty of Engineering, University of Porto, 4200-465 Porto, Portugal

**Keywords:** 3D printing, cement-based materials, waste valorization, sustainability, circular economy

## Abstract

Three-dimensional Cementitious materials Printing (3DCP) is a cutting-edge technology for the construction industry. Three-dimensional printed buildings have shown that a well-developed automated technology can foster valuable benefits, such as a freeform architectural design without formworks and reduced human intervention. However, scalability, commercialization and sustainability of the 3DPC technology remain critical issues. The current work presents the ecological fragility, challenges and opportunities inherent in decreasing the 3DCP environmental footprint at a material level (cementitious materials and aggregates). The very demanding performance of printable mixtures, namely in a fresh state, requires high dosages of cement and supplementary cementitious materials (SCM). Besides the heavy carbon footprint of cement production, the standard SCM availability might be an issue, especially in the longer term. One exciting option to decrease the embodied CO_2_ of 3DCP is, for example, to incorporate alternative and locally available SCM as partial cement replacements. Those alternative SCM can be wastes or by-products from industries or agriculture, with no added value. Moreover, the partial replacement of natural aggregate can also bring advantages for natural resource preservation. This work has highlighted the enormous potential of 3DCP to contribute to reducing the dependence on Portland cement and to manage the current colossal wastes and by-products with no added value, shifting to a Circular Economy. Though LCA analysis, mixture design revealed a critical parameter in the environmental impact of 3DCP elements or buildings. Even though cement significantly affects the LCA of 3DCP, it is crucial to achieving adequate fresh properties and rheology. From the literature survey, mixtures formulated with alternative SCM (wastes or by-products) are still restricted to rice husk ash, Municipal Solid Waste ashes and recycled powder from construction and demolition wastes. Natural aggregate replacement research has been focused on recycled fine sand, mine tailing, copper tailing, iron tailing, ornamental stone waste, recycled glass, crumb rubber, rubber powder and granules, recycled PET bottles and steel slag. However, flowability loss and mechanical strength decrease are still critical. Research efforts are needed to find low-carbon cement replacements and mix-design optimization, leading to a more sustainable and circular 3DCP while ensuring the final product performance.

## 1. Introduction

The recent forecast has stressed that buildings and construction consume about 40% of the natural resources, 70% of electrical power, 12% of potable water, generate 45–65% of landfill waste and 48% of Greenhouse Gases (GHG) emissions [1,2,3,4]. In Europe, construction is an economic sector which directly employs 18 million people and creates close to 10% of the European Union Gross Domestic Product (GDP). Thus, construction is key to a smarter, sustainable, and inclusive society while contributing to job creation and economic growth; essential pillars of the 2030 Agenda for Sustainable Development Goals (SDG) [5,6]. 

**Figure 1:**
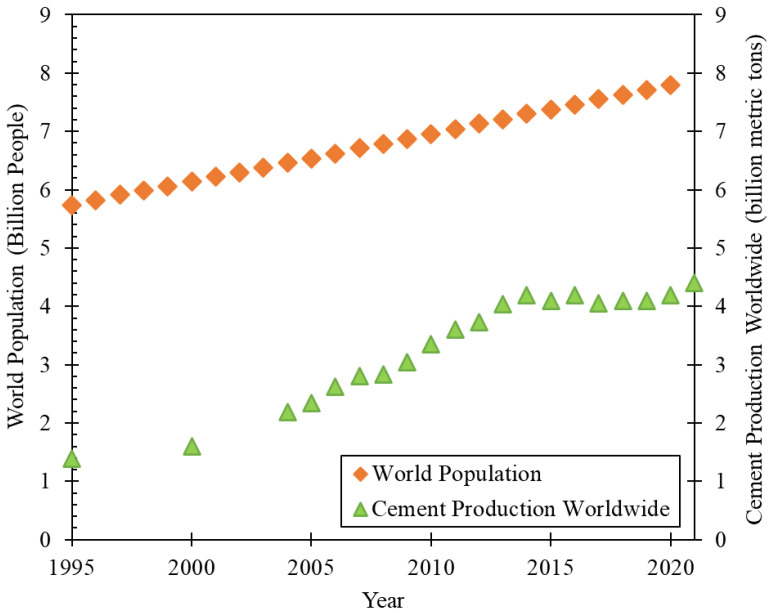
World population (data source [10]) and cement production worldwide (data source [11,12]).

Concrete, a composite material mostly constituted of cement, aggregates and water, is the second-most used material in the world [7]. Indeed, no other material is expected to meet the demands of the world’s population for housing and infrastructure [8,9]. Even though concrete has a lower embodied CO_2_ than other building materials (such as asphalt or bricks), humanity’s dependence on this composite material has brought some concerns. This is mainly because the basic raw materials for concrete manufacture are non-renewable natural resources, and Portland cement production has a heavy ecological footprint. However, as seen in Figure 1, cement demand is expected to increase with population growth in the coming years [8,9]. 

On the other hand, the construction industry is dealing with new challenges to increase productivity following more technologically advanced, sustainable and circular principles. Historically, construction has been a labour-intensive industry with low technological usage, a marked lack of innovation, and conservative, which has resulted in long stagnation [7,13]. The main barriers to construction upgrades are the high number and fragmented stakeholders, insufficiently qualified workforce and excessive manual and heavy labour. Recently, the COVID-19 pandemic had profound consequences on labour work. At the beginning of 2020, the construction industry was severely affected by unscheduled closures of job sites, a lack of materials and multiple issues in the supply chain, and suspended projects. Productivity, labour workforce and cash flow negatively impacted the construction industry and many other businesses. 

Meanwhile, construction digitalization, a 3D printing construction method, has earned relevance due to the potential to deal with health crises [14] and, more recently, war crises [15]. According to the predictions of Markets and Markets, 3D print construction methods can decrease waste by 30–60% and time by 50–70% in construction [16]. Indeed, using 3D printing technology in the construction industry can reduce the need for on-site workforce since continuous project processing is through remote control via Building Information Modeling tools and Artificial Intelligence. Therefore, in addition to increasing productivity, 3D construction printing creates high-end technology-based jobs and solves the existing limitations of the labour workforce in this industry [17]. Improvement in worksite safety and reduced work accidents is expected due to the lower dependency on human labour and consequently less risks [17,18]. 

The most widespread 3D printing technology in the construction industry is 3D cementitious materials printing (3DCP), usually mortars. Cement-based building elements, or particular architectural elements, can be manufactured through 3D printing [17], being designed on a computer and then extruded from a nozzle of a 3D printer which manufactures a physical object by automatically depositing successive layers [19,20]. In addition, 3DCP does not require formwork and/or temporary structures, which are responsible for a considerable amount of waste generation (around 23% of total) [17,18], materials consumption, time and labour costs (from 35 to 60% of global costs) [17,18,21]. Moreover, 3DCP allows more architectural freedom due to no need for formwork [7,22,23]. The potential of 3DCP seems enormous, and its implementation is increasingly a reality, capable of revolutionizing the construction industry. 

## 2. Research Significance and Objectives

**Figure 2:**
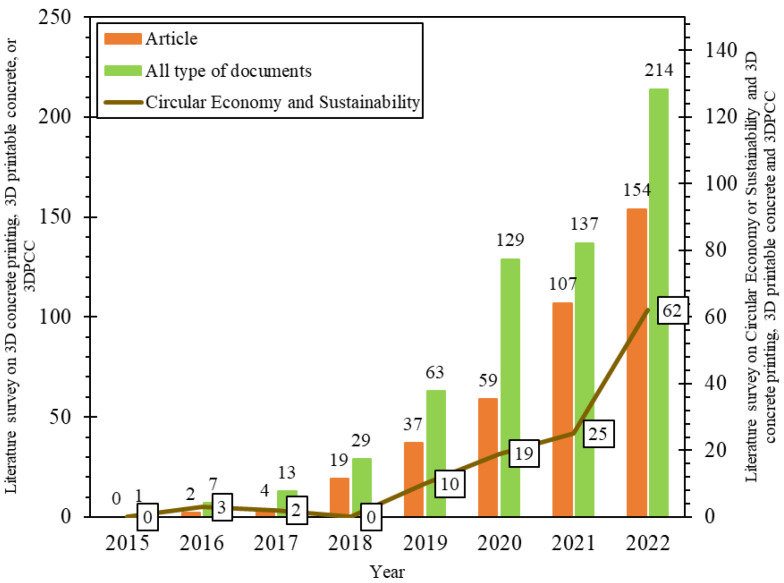
Number of documents published in English related to “3D concrete printing” or “3D printable concrete” or “3D printable cement-based composites” and literature on this topic related to “Circular Economy” or “Sustainability” (Data collected from Scopus in 26 December 2022).

Interest in 3DCP has been noticed since 2015, and available literature has increased substantially over the last four years, as depicted in Figure 2. Several institutions, universities, and companies worldwide have exhibited digitally manufactured prototypes of structural components, furniture, and full-scale show-off buildings [22,23,24,25,26,27]. However, scalability, commercialization and sustainability of the 3DCP technology remain critical issues [28].

Developing 3D Printable cement-based composites (3DPCC) is a main pillar for successful 3D printing structures and buildings. The mixture design must comply with multi-performance requirements at all stages of the 3D printing process, namely [13,29]:

1. pumpability, suitability for moving under pressure in the pipe;
2. extrudability, the ability to pass through the nozzle under pressure, printing a filament without disruption;
3. buildability, ability to print successive layers of 3DPCC maintaining the shape of layers without deformation or collapse;
4. printing open time, a workable time for the printing process from mixing to deposition, taking into account factors such as scale and print speed.

Rheology is the predominant parameter to control printing requirements (especially pumpability, extrudability and buildability) of 3DPCC. Even though rheology mostly depends on materials, it also depends on the printer apparatus characteristics, such as pumping distance and pump type, shape and size of the nozzle, and print speed [13,30], which are not discussed in the current work. 

To ensure printable performance, 3DPCC mixture design generally requires finer materials (cement and supplementary cementitious materials) [31]. In addition, higher dosages and multiple chemical admixtures (superplasticiser, Viscosity Modifying admixture (VMA), Hydroxyethylmethyl Cellulose HEMC) are usually needed. Those features increase CO_2_ emissions and energy consumption more than traditional casting concrete. Therefore, from a materials science view (and not only), 3DCP faces significant challenges. It is necessary to solve the problem of material clogging to required engineering printing properties while keeping robust, sustainable, and circular cementitious-based mixtures. 

The binder (namely Portland cement) is, by far, the main constituent of 3DPCC, which raises serious concerns. An exciting sustainability approach can be pursued by partially replacing a significant fraction of the cement with locally available SCM or alternative materials, such as waste or by-products with no added value. On the other hand, the partial replacement of natural aggregate by alternative sources, such as recycled aggregates, can also reduce the embodied CO_2_ of 3DCP and contribute to natural resource preservation. Thus, studying locally available wastes or by-products and incorporating these unconventional materials is also an opportunity to create synergies between different industries and boost their circularity [23,32].

However, to date, the sustainability of 3DCP, primarily by reducing the Portland Cement (PC) content, is still limited and has not been adequately discussed and summarized. The current work provides a comprehensive analysis of raw materials currently used in the mixtures design of 3DCP. The review explores standard and unconventional materials previously studied in 3DCP to obtain more sustainable mixtures, especially the potential of waste and by-products. Additionally, a comprehensive review of the influences of different SCM on the printability of cementitious materials is addressed. The aim is to understand the emerging trends in literature identifying challenges in 3DCP, alternatives to reduce cement and/or natural aggregate demand, and opportunities to reduce the environmental impact of 3DCP and improve its sustainability and circularity. As such, Section 3 presents the main findings of the sustainability evaluation of 3DCP via Life Cycle Analysis (LCA). Afterwards, Section 4 highlights and discusses the mixtures design of 3DPCC available in the literature based on statistical analysis. Section 5 describes, in detail, the raw materials used in 3DPCC formulations, including conventional and unconventional materials, and their effect on the final composite performance. Finally, Section 6 summarises the main conclusions of future research needs.

## 3. Sustainability Analysis

The Life Cycle Assessment (LCA) is a powerful tool to support the development of a Circular Economy [33]. Still, the environmental impact of 3DCP is poorly explored. 

At the material level, mixture design has an important role in the environmental impact of 3DCP and is the key to improving its sustainability. Cement significantly affects the LCA of 3DCP [34], but higher quantities of cement are crucial to achieving adequate printability properties. Cement content is more critical than cement type from an environmental point of view. The LCA of 3DPCC using CEM I 32.5, CEM I 42.5, and CEM I 52.5 for 3DCP was similar, with a variation of about ±2% [35]. In this way, SCM are feasible options to partially replace PC and decrease the ecological footprint of 3DCP, such as silica fume [32,36], fly ash [32,37,38], ground granulated blast furnace slag (GGBFS) [36,37], and limestone calcined clay [32]. Limestone calcined clay (LC2) as SCM (40 and 50% wt.) decreased GHG emissions between 41.6 and 50.2% and energy consumption between 38.2 and 45.2%, compared to 3DPCC without SCM [39]. In addition, secondary aggregates and aggregates from waste or by-products can also reduce the environmental impact of 3DCP, especially if given preference to locally available alternatives without upcycling strategies. Total replacement of natural aggregate by recycled aggregate reduced global warming (−2.5%), acidification (−5.9%), photochemical pollution (−37.5%) and eutrophication (−13.0%) in 3DPCC [34]. 

Other studies performed LCA at a structural scale comparing 3DCP with conventional construction methodologies [32,40]. Alhumayani et al. [41] compared a 1 m^2^ 3DCP wall with a thickness of 40 cm with an internal pattern filament and a conventional concrete wall including columns, beams and blocks. In the LCA cradle-to-site approach, a 3DCP wall showed an overall environmental improvement of 24.0%; namely, better environmental performance for stratospheric ozone depletion (−11%), fine particle matter (−24%), marine eutrophication (−47%), land use (−94%), mineral resource scarcity (−60%) and water use (−15%) categories. Despite an overall improvement, the conventional concrete wall showed lower global warming (−27%) [41]. Ali [42] employed cradle-to-gate LCAs of materials and processes in 3D printed and conventional buildings for a terraced house with a floor area of 60 m^2^. The author concluded that 3D printing has a lower environmental impact, while conventional construction presented higher damage to human health (+78%), ecosystem quality (+76%), climate change (+80%) and resources (+75%) [42]. Weng et al. [43] compared the environmental performance of prefabricated bathroom units (1620 × 1500 × 2800 mm^3^) produced using 3D printing and pre-casting. The authors concluded that 3D printing technology reduced CO_2_ emissions (−85.9%) and energy consumption (−87.1%). In addition, using 3D printing technology resulted in a lighter self-weight structure (−26.2%) and increased productivity (+48.1%) [43].

**Figure 3:**
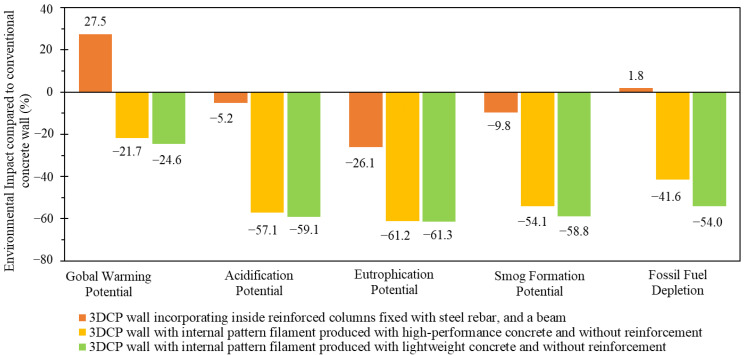
Environmental impact (%) by category of three 3DCP scenarios compared with the reference value of construction of the conventional wall (data source [35]).

In a more detailed study, Mohammad et al. [35] used a cradle-to-gate analysis to compare different wall solutions produced through conventional construction (used as reference) and 3D printing technology (three different scenarios). The conventional wall consists of hollow concrete masonry units, two reinforced concrete columns and a reinforced concrete beam. The three 3DCP scenarios included, in brief: (i) a 3DCP wall with the reinforcement concrete structural system; (ii) a hollow wall with internal pattern filament produced with high-performance concrete and without reinforcement; and (iii) a hollow wall with internal pattern filament produced with lightweight concrete and without reinforcement. The lightweight concrete mixture design included expanded perlite aggregate (particle size < 4 mm) and high-performance concrete using a river sand aggregate (particle size < 2 mm). The remaining constituent materials, such as Portland cement, fly ash, micro silica, water, polypropylene microfiber, superplasticizer and accelerator, were the same and kept in equal proportions [35]. Figure 3 presents the environmental impact (%) of three 3DCP scenarios by category compared to the conventional wall (reference value of 0%). As can be seen, 3DPC using lightweight concrete without reinforcement presented the best environmental performance, followed by high-performance 3DCP without reinforcement. Lightweight 3DPC (see Figure 3, green bars) offered the lowest global warming potential (−24.6%), acidification potential (−59.1%), eutrophication potential (−61.3%), smog formation potential (−58.8%), and fossil fuel depletion (−54.0%) [35]. The environmental performance of the 3DCP wall with the reinforcement concrete structural system was the worst compared to the other 3DCP solutions (see Figure 3, orange bars) and even presented higher global warming potential compared to the conventional wall solution used as reference.

## 4. Mixtures Proportions 

### 4.1. Data Collect and Analysis

Compared to conventional concrete, 3DCP has a different mixture design and fresh and hardened properties. Conventional concrete has a higher workability and lower slump. On the other hand, 3DPCC requires lower workability and a rapid setting time [17]. In general, the 3DPCC mixture design relies on (i) lower aggregate content, in which aggregate-to-binder weight ratio can be lower than 1.0; (ii) a higher amount of fine powder materials (cement and supplementary cementitious materials); (iii) different types of admixtures.

**Figure 4:**
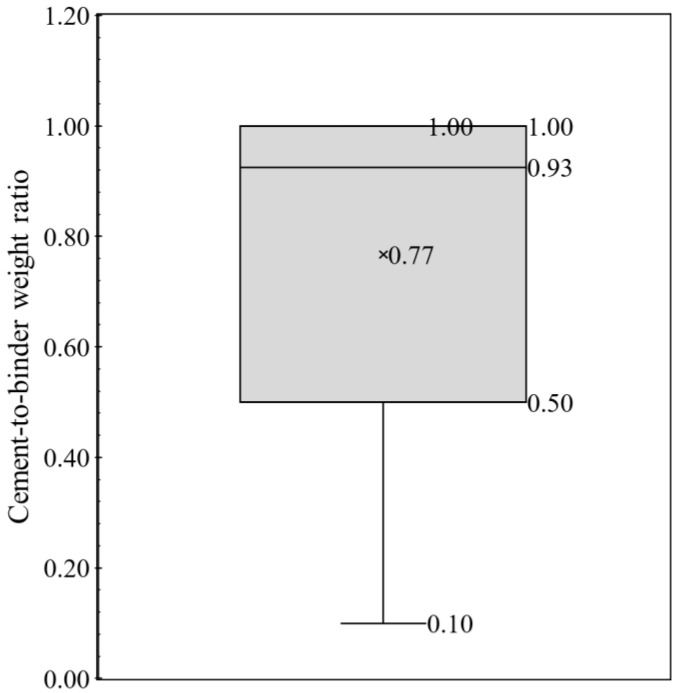
Whiskers box of cement-to-binder weight ratio for 157 mixtures design based on literature survey (data source: [30,44,45,46,47,48,49,50,51,52,53,54,55,56,57,58,59,60,61,62,63,64,65,66,67,68,69,70,71,72,73,74,75,76,77,78,79,80,81,82,83,84,85,89,90,91,92]).

**Figure 5:**
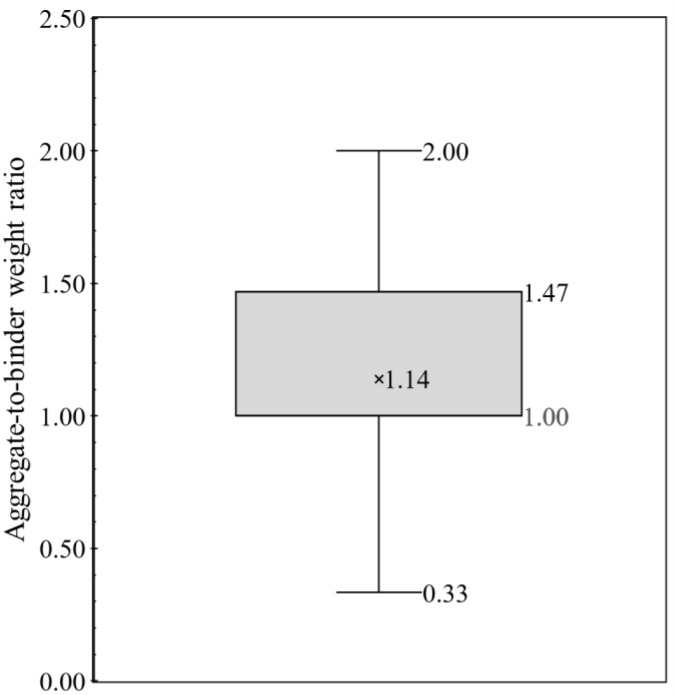
Whiskers box of aggregate-to-binder weight ratio for 157 mixtures design based on literature survey (data source: [30,44,45,46,47,48,49,50,51,52,53,54,55,56,57,58,59,60,61,62,63,64,65,66,67,68,69,70,71,72,73,74,75,76,77,78,79,80,81,82,83,84,85,89,90,91,92]).

**Figure 6:**
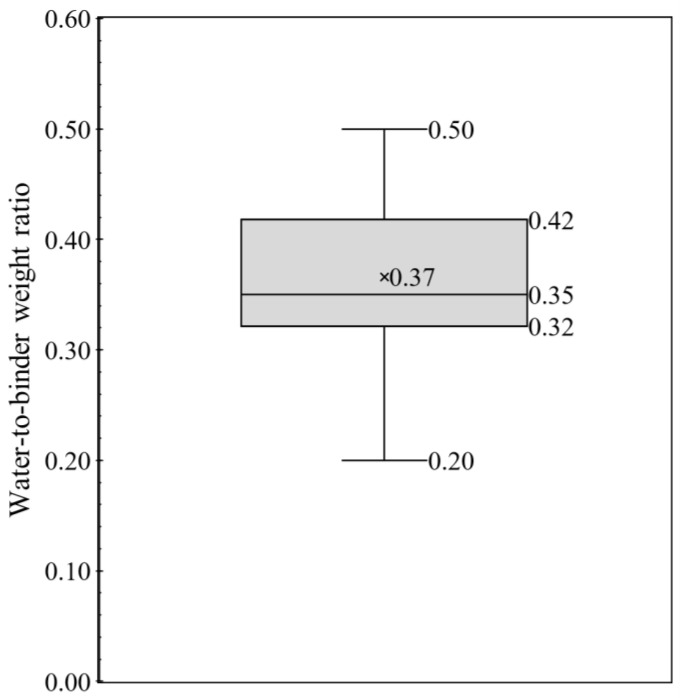
Whiskers box of water-to-binder weight ratio for 157 mixtures design based on literature survey (data source: [30,44,45,46,47,48,49,50,51,52,53,54,55,56,57,58,59,60,61,62,63,64,65,66,67,68,69,70,71,72,73,74,75,76,77,78,79,80,81,82,83,84,85,89,90,91,92]).

The current work focused on powder constituent materials (cement and aggregates) of 3DPCC and has conducted an exhaustive review to collect mixture proportions. From the literature survey, 157 3DPCC mixture proportions were found in 47 references [34,44,45,46,47,48,49,50,51,52,53,54,55,56,57,58,59,60,61,62,63,64,65,66,67,68,69,70,71,72,73,74,75,76,76,77,78,79,80,81,82,83,84,85,86,87,88]. Even though 3DCP has been under scrutiny recently, the actual values, in terms of weight (kg/m^3^) or volume (m^3^/m^3^), of each constituent material employed in the mixture formulations were not always available in the literature; sometimes only some mixture design ratios were provided. Thus, the lack of mix formulations data constrained the mixtures design analysis. To overcome this barrier, the mixture design analysis is presented here through typical concrete mixture ratios: cement-to-binder weight ratio, aggregate-to-binder weight ratio and water-to-binder weight ratio. A statistical analysis of those mixtures ratios was performed using whiskers boxes illustrated in Figure 4, Figure 5 and Figure 6, respectively. Each whisker box presents the 25th percentile, 75th percentile, minimum and maximum, average and median values of cement-to-binder weight ratio (Figure 4), aggregate-to-binder weight ratio (Figure 5) and water-to-binder weight ratio (Figure 6) concerning 157 3DPCC mixture proportions found in the literature review. 

As seen in Figure 4, the mean and median value of cement-to-binder weight ratio corresponds to 0.77 and 0.93, respectively. In addition, most mixture designs (≥75%) maintain cement incorporation superior to 50% wt. in binder fraction. The maximum value and 75th percentile value correspond to 1.00, and refer to 3DPCC mixtures using only cement as a binder. This means the 3DPCC mixture employed high cement dosages with lower SCM content. Another interesting indicator is the aggregate-to-binder weight ratio, see Figure 5, in which average and median values correspond to 1.14 and 1.00, respectively, being the median value equal to 25th percentile. This translates the high dependence of 3DPCC on binder fraction. Regarding the water-to-binder weight ratio, see Figure 6, a wide range was found between 0.20 to 0.50. The average and median water-to-binder weight ratio correspond to 0.37 and 0.35, respectively. Moreover, it was found that the admixtures incorporation of 3DPCC is recurrent; namely, combining two admixtures, such as superplasticizers, Hydroxypropyl Methylcellulose (HPMC), polycarboxylate-based high-range water reducing admixture, Viscosity Modifying Admixture (VMA), and nano-clay and sodium gluconate.

### 4.2. Mixtures Analysis Based on Constituent Materials

**Figure 7:**
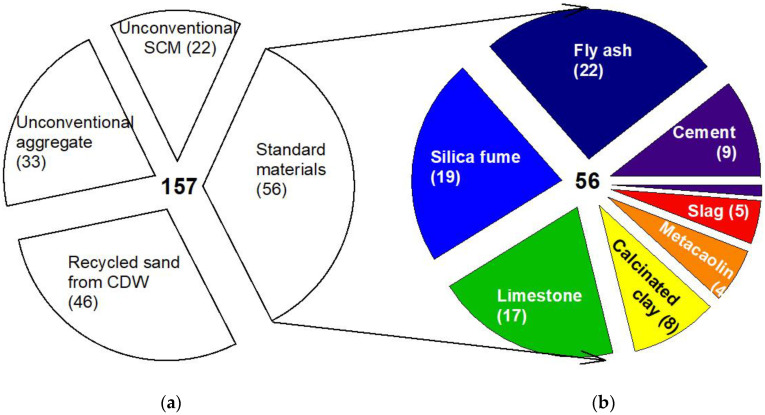
(**a**) 3DPCC constituent materials sources overview; (**b**) 3DPCC standard materials used as binder constituents.

The data set (157 3DPCC mixtures) highlighted four different types of mixtures based on constituent materials, as depicted in Figure 7a: 56 mixtures formulated exclusively with standard materials (cement, standard SCM and natural aggregates), 22 mixtures with unconventional SCM (from wastes or by-products) as partial cement replacement, 33 mixtures with unconventional aggregate as partial and total natural aggregate replacement, and 46 with recycled sand from Construction and Demolition wastes (CDW) as partial and total natural aggregate replacement. From the 56 mixtures composed of standard materials, 9 mixtures employed Portland Cement exclusively as binder, and the remaining were binary or ternary blends, see Figure 7b. A predominance was observed using Fly Ash (FA), incorporated in 22 mixtures, in which average and median values of FA-to-binder weight ratio were 0.47 and 0.48, respectively. Silica Fume (SF) has also been studied in 19 3DPCC mixtures with average and median values of SF-to-binder weight ratio of 0.09 and 0.05, respectively. Limestone filler (LF) was used in 17 3DPCC mixtures with mean and median values of LF-to-binder weight ratio of 0.23 and 0.20, respectively. Other standard SCM were less representative. Eight mixtures were found with calcined clay (average and median values of calcined clay-to-binder weight ratio 0.43 and 0.40, respectively), five mixtures with Ground Granulated Blast Furnace Slag (GGBFS) (average and median values of GGBF-to-binder weight ratio 0.74 and 0.80, respectively), four mixtures with metakaolin (average and median values of metakaolin-to-binder weight ratio 0.09 and 0.10, respectively) and one mixture with limestone-calcined clay (LC2) (LC2-to-binder weight ratio 0.30).

**Figure 8:**
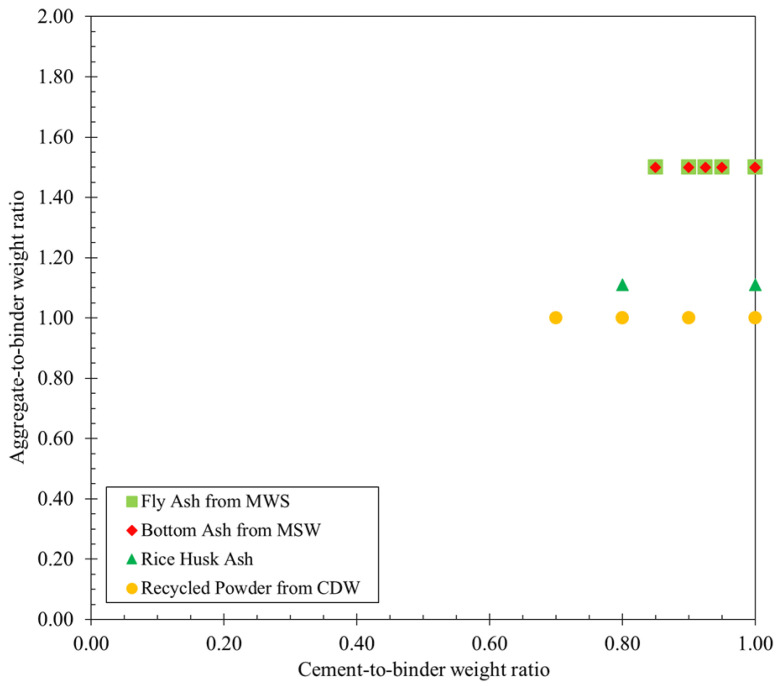
Cement-to-binder weight ratio and aggregate-to-binder weight ratio in 22 3DPCCmixtures incorporating unconventional SCM as cement replacement (data source: [60,61,62,63,64]).

Regarding the unconventional SCM (found in 22 3DPCC mixtures), the most studied cement substitute was recycled powder from CDW, incorporated in seven mixtures with a mean and median waste-to-binder weight ratio of 0.16 and 0.14, respectively [62,63,64]. Fly and bottom ashes from Municipal Solid Waste (MSW) were also studied as partial cement replacement on 3DPCC mixtures with replacement ratios of 0.05, 0.08, 0.10, and 0.15 [60]. In addition, a mixture design with rice husk ash as SCM was studied with a waste-to-binder weight ratio of 0.20 [61]. Figure 8 depicts the cement-to-binder weight ratio and aggregate-to-binder weight ratio obtained from 22 mixtures design using unconventional SCM. As can be seen, the range of cement replacement by unconventional SCM is between 0% and 30% by weight, and the aggregate-to-binder weight ratio varies between 1.00 and 1.50. Figure 8 also highlights that the mixtures incorporating MSW ashes allowed a higher aggregate content in 3DPCC (aggregate-to-binder weight ration 1.50), followed by RHA (aggregate-to-binder weight ration 1.10). The 3DPCC mixtures with recycled powder from CDW as partial cement replacement employed higher binder content (aggregate-to-binder weight ration 1.00, see Figure 8). A detailed characterization of conventional and unconventional SCM effects on composite properties is presented in Section 5.2.

**Figure 9:**
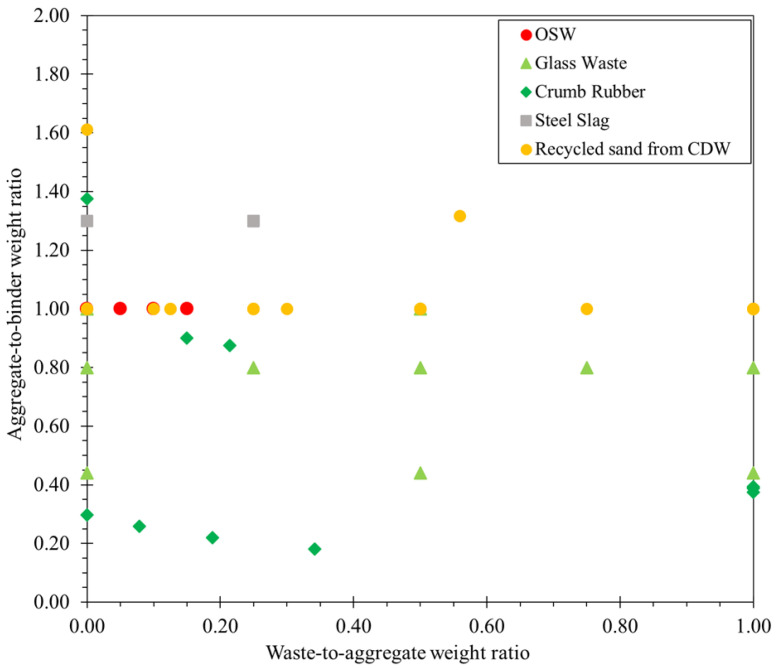
Aggregate-to-binder weight ratio and waste-to-total aggregate weight obtained from 3DPCC mixtures using unconventional aggregates (data source: [65,66,68,70,71,72,74,75,76,77,78,79,80,81,82,83,84,85].

In terms of alternative sources to natural aggregates, the valorization of waste materials, such as copper tails, iron tails, and recycled aggregate [34,76,86,87], was explored by a few authors. Figure 9 shows the aggregate-to-binder weight ratio and unconventional aggregate-to-total aggregate weight ratio of 3DPCC mixtures using unconventional aggregates, namely Ornamental Stone Waste (OSW), Glass Waste (GW), crumb rubber, steel slag, and recycled sand from CDW.

## 5. Powder Constituent Materials: Characterization and Effect

The adequate selection and characterization of constituent raw materials will have a crucial role in the performance of 3DPCC both at material and structural levels [13,88]. Such selection has to consider the performance requirements at engineering, aesthetics, economic and environmental levels. 

In general, constituent materials employed in 3DPCC are the same as other cement-based materials, which are cement, SCM, fine aggregates, and admixtures, but in different proportions [93]. As discussed in Section 4, 3DPCC requires a large quantity of cement, SCM, and a combination of different admixtures [13,17]. Mixtures are usually designed in the scale of mortar with limited aggregate fraction and particle size generally inferior to 2 mm [93,94]. Characteristics of 3D printing equipment and the dimensions of the nozzles are the main factors conditioning the particle size of 3DPCC [13]. The following sections discuss the current standard and unconventional constituent materials employed to produce 3DPCC.

### 5.1. Cement

**Figure 10:**
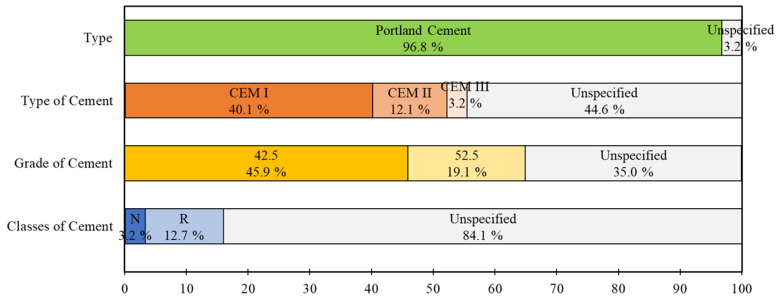
Type, type of cement, grade of cement, and class of cement for 157 3DCP mixtures design (%) (data source: [30,44,45,46,47,48,49,50,51,52,53,54,55,56,57,58,59,60,61,62,63,64,65,66,67,68,69,70,71,72,73,74,75,76,77,78,79,80,81,82,83,84,85,89,90,91,92]).

Hydraulic cements, constituted mainly of calcium silicates and aluminates, are the most exciting construction and civil engineering binders. Portland cement (PC) is the most widely used cement [95]. Even though alternative cements have emerged, as sulfur-based cements [96], limestone calcined [97], clay cements, calcium aluminate cements [98] and geopolymers [99,100], the main ingredient for 3DPCC is cement, namely, PC, which is the focus of the current work. According to EN 197-1, 27 types of cements are certified in Europe, even though each country has only some types available depending on raw resources availability. Figure 10 shows the characteristics of cement used in 157 3DPCC mixtures design found in the literature survey. Once again, the lack of information has constrained the data analysis. Available data show that the researchers mainly employed Portland Cement type I (40.1% of 3DPCC mixtures) followed by CEM II (12.1% of 3DPCC mixtures), and a small slice used CEM type III. Regarding strength class, most researchers have been using class 42.5. 

The paste volume and composition, i.e., cement type and SCM, strongly influence the 3DPCC behaviour both in fresh and hardened states. It is recognised that a high paste volume (>50%) and low dosage of aggregates content (<50%) allow better pumpability and extrudability at a constant fresh consistency [101] and achieve a smoother surface for 3DCP elements. However, a higher paste volume can increase or decrease the compressive and flexural strengths of 3DPCC, depending on the constituent materials. Partial PC replacement by SCM at a constant paste volume, such as FA and micro silica fume, may reduce the early compressive strength and improve interlayer-bond strength [102]. Thus, paste volume and composition are effective parameters for optimizing the properties of 3DCP in fresh and hardened states [89,103]. 

### 5.2. Supplementary Cementitious Materials

Besides reducing the costs and environmental impact of 3DPCC [94], some SCM may provide significant physical advantages, such as granular packing density, cohesion, flow consistency, decrease in the heat of hydration and crack formation [17]. Pozzolanic SCM can also react with calcium hydroxide and provide additional calcium silicate hydrates (CSH), which may improve mechanical properties and durability [104]. 

**Figure 11:**
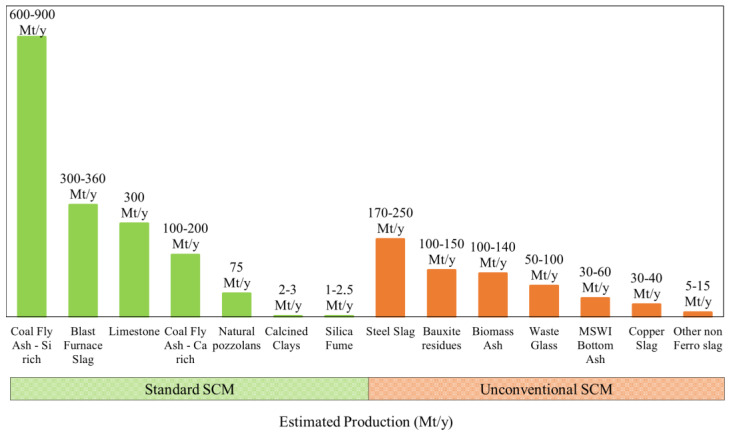
Worldwide availability of standard and unconventional SCM (Mt/y) that can be incorporated in cement-based materials (data source [110]).

Figure 11 shows the worldwide availability of standard and unconventional SCM that can be used in cement-based materials. Standard SCM can be classified as quasi-inert materials, usually called fillers (type I additions) or materials with latent hydraulic or pozzolanic properties (type II additions). The most widely used type II standard SCM are GGBFS and FA. Nonetheless, the availability of GGBFS and FA is relatively reduced, attending to the actual demand of cement production and may be an issue considering a long-term vision. Currently, cement factories and concrete production industries already consume more than 90% of the available GGBFS. Although the worldwide availability of FA is about 600 to 900 Mt/year, only about a third of FA is used in cement and concrete industries as a consequence of the inconstant properties of FA that affect quality and suitability in cement-based products [6]. Moreover, adequate FA availability is aggravated by reducing coal for energy production to decrease carbon emissions and reach decarbonization. The benefits of using SF in cement-based materials are also well known; however, it is expensive and may not be readily available in some countries. Limestone filler, an almost inert SCM, is currently widely used in European countries, namely in composite cements (CEM Type II), according to EN 197-1 [105]. LF offers technical advantages in cement-based materials, such as providing better workability, density, and compactness and decreasing the permeability, capillarity, exudation and cracking risk. PC hydration can be improved by incorporating fillers, since fillers act as nucleation centres [7], especially in dosages up to 10% as cement replacement [12,13,106]. However, dosages higher than 10% may increase porosity and decrease strength [10,107,108]. Moreover, LF is derived from a non-renewable natural resource, and its production consumes energy to obtain the required standard properties, namely fineness. 

As such, many limitations may arise if standard SCM are one of the main constituents of 3DCP in a long-term application. Moreover, the worldwide availability might be highly variable across countries. For instance, there is a scarcity of FA and GGBFS in Portugal, and concrete producers mainly use LF as SCM or composite cement type II, according to EN 197-1. 

Binary, ternary, and quaternary cementitious blends with SCM have been studied [109], but each SCM impacts the fresh and hardened properties of the 3DPCC final product in a specific way [101]. The following sections present the SCM found in the literature survey to produce 3DPCC, both standard SCM (Section 5.2.1) and unconventional (Section 5.2.2).

#### 5.2.1. Standard SCM

SCM with pozzolanic properties has economic and environmental advantages on the final cementitious material products, as well as engineering benefits such as slower release of hydration heat and development of strength, refinement of the pores, and improved strength and water tightness of the concrete. However, the efficiency depends on its pozzolanicity. Dedicated standards are available to certify and guarantee the quality of standard SCM and are summed up in Table 1, considering: (i) the European standards for silica fume EN 13263-1; (ii) coal fly ash according to EN 450-1; (iii) slag according to EN 15167-1; (iv) the Portuguese standard NP 4220 for pozzolanic materials other than the ones considered in European standards; and, (v) the American standard for fly ash and raw or calcined natural pozzolan ASTM C 618. 

As can be perceived from Table 1, SCM are mainly composed of silica (SiO_2_), alumina (Al_2_O_3_), iron oxide (Fe_2_O_3_), and other oxides in minor proportions, such as calcium oxide (CaO). Moreover, some limits are imposed in terms of compounds which may have a detrimental effect on concrete or steel reinforcement, such as chlorides (EN 450–1, EN 151671–1 and NP 4220 define 0.1% as maximum chloride content), sulphates (limit of 3.0% for SO_3_ specified in EN 450 for the use of coal fly ash), alkali content and LOI (the total organic carbon content presents maximum limits of 5%, 7%, and 9% for Category A, B and C fly ashes, respectively, according to EN 450–1, and 10% for class N and 6% for the remaining classes according to ASTM C 618).

The literature review showed that the standard SCM used as partial cement replacement in 3DPCC are FA, followed by SF, LF, calcined clay, and metakaolin, as presented in Section 4. It follows some discussion of the effect of the aforementioned SCM on 3DPCC. In addition, the main physical and chemical characterization of SF, LF, calcined clay, metakaolin, and slag properties used on 3DPCC are summarized in Table 2 and Table 3, respectively.

FA is a by-product of coal combustion during electricity power production in thermal power plants [111], and its incorporation has multiple benefits, including, among others, durability, shrinkage, porosity, and decreasing heat of hydration. The main physical and chemical FA properties used on 3DPCC are summarized in Table 2 (lines 1 to 3) and Table 3 (lines 1 to 5), respectively. As expected, depending on the source, FA may present differences. Cement replacement with low (around 20% wt. of binder) [30,51,52,112] or high quantities (around 70% wt. of binder) [46,90,113,114] of FA has been studied by several authors to fulfil printing requirements and reduce cement amount of 3DPCC mixtures. FA as a cement replacement (from 50 to 80% wt.) reduces frictional forces between PC particles of cementitious matrices in mixtures with a constant w/b of 0.45 [46]. This SCM modified the fresh properties of 3DPCC and decreased static yield stress and viscosity, resulting in an intensification of bottom layer deformation during loading caused by the succeeding printed layers. Despite these disadvantages, it improved the extrudability of 3DPCC [46]. For 3DPCC with a high volume of FA (70%) and PC, nano attapulgite clay (from 0.1 to 0.5% wt. binder) was found to be a viable additive to enhance the printability of the mixtures [90]. This nano-material improves buildability, and printed layers presented a lower deformation since yield strength increases and viscosity were not significantly affected [90]. Klyuev et al. [113] studied ternary binders with PC (from 30 to 70% wt. binder), FA (from 60 to 20% wt. binder), and crushed quartzite sandstone (10% wt. binder) and observed the request of adding superplasticizer and adjusting water content. Increasing FA and decreasing PC causes an increase in initial and final setting time, more emphasized for higher percentages of FA [113]. However, as expected, the mechanical properties of 3DPCC with a high FA content were not so attractive. The best results were determined for 3DPCC with 20 and 30% wt. FA. A 3DPCC ternary blend of 70% PC, 20% FA and 10% crushing quartzite sandstone enhanced 23.1% and 51.9% on compressive and flexural strength at 28 days, respectively, compared to mixtures exclusively composed by CEM I 42.5N [113]. Recent research on the influence of FA replacement with different SCM has also emerged in the literature [52,114]. In ternary binders with 70%wt. PC, FA and aggregate micro fines (up to 12% FA replacement), 3DPCC showed adequate workability with a decrease in flowability and extrudability, but with an increase in early strengths and shape stability caused by the irregularity of aggregate micro fines [52]. Another SCM used as FA replacement was LF (from 0 to 15% wt. binder) in ternary mixtures with 70% FA and 30% PC [114]. Dey et al. [114] developed extrudable and buildable 3DPCC and observed an increase in yield stress with increased LF content. 

Metakaolin produced from the calcining process of kaolinitic clay at temperatures between 450 °C and 900 °C was also incorporated in 3DPCC due to its high pozzolanic properties [115]. Metakaolin employed in 3DPCC was mainly composed of silica (52.8–58.18% wt.) and aluminium oxide (23.33–42.00% wt.), as can be seen in Table 3 (lines 6 and 7). However, it is expensive and produced in limited quantities. Duan et al. [53] verified that between 5 and 10% wt. metakaolin as cement replacement significantly influenced rheological properties; static yield stress increased 145.8 and 285.5%, respectively, dynamic yield stress improved 61.9% and 129.4%, respectively, and viscosity increased 48.4 and 49.2%. Percentages of metakaolin between 5 and 10% wt., enhance layer stability, achieving a higher number of printed layers and height [53]. Barbosa et al. [54] also observed an increase in viscosity caused by replacing 10% wt. of SCM by metakaolin in 3DPCC with 60% PC and 40% LF. Metakaolin as LF replacement requires a higher superplasticizer dosage and water to decrease viscosity and increase fluidity, reaching printable mixtures without defects and with better compressive and flexural strength and interlayer adhesion strength [54].

Calcined clay has also been used in 3DPCC along with LF to decrease PC content and improve fresh and hardened properties [44,55,56,116]. The main properties of calcined clay employed in 3DPCC are presented in Table 2 (lines 6 to 9) and Table 3 (lines 8 to 11). As can be seen, calcined clay is mainly constituted by silica (47.1–55.1% wt.) and aluminium oxide (38.4–50.6% wt.), while LF is calcium oxide (39.6% wt.). As previously mentioned, LF is a standard SCM widely used in cementitious materials and is a result of limestone production in quarries, followed by a grinding treatment [115]. The influence of cement replacement [55], graduation of calcined clay [56], superplasticizer dosage [55] and viscosity-modifying admixture [44] in ternary binders composed of PC, LF and calcined clay has been studied. The reduction of PC (from 100% to 10% wt.) and increase of LF and calcined clay decreased flowability [55]. High-grade calcined clay with 95% metakaolin positively affected buildability by increasing cement hydration and accelerating reactions compared to low-grade calcined clay (50% metakaolin) [56]. On the other hand, high-grade calcined clay also reduced the printability window, which can be a disadvantage in the printing process [56]. Chen et al. [44] determined the optimal VMA dosage of 0.24% wt. of binder in terms of shape stability, buildability and early strength and mechanical properties for 3DPCC with 40% PC, 20% LF, and 40% low-grade calcined clay by weight.

SF is an ultrafine and very reactive powder, a by-product from silicon and ferrosilicon alloy production in an electric arc furnace at high temperatures. SF is predominantly amorphous silicon oxide (93.96–96.8%wt.) [111,115,117], and contains extremely fine and spherical particles with dimensions inferior to 1µm, as summarized in Table 2 (lines 14 to 17) and Table 3 (lines 15 to 18). SF acts as a filler, filling voids between cement particles and as a pozzolan which reacts with calcium hydroxide, causing CSH gel formation [118]. This SCM is a noble option to partially replace cement up to 10% wt. and increase yield stress and viscosity of mixtures; it keeps printing quality, improving the shape stability of layers and minimizing deformation of bottom layers [30,46,57]. Lower percentages of SF (2.0, 2.5 and 5.0% wt.) may be a way to increase yield stress and viscosity and decrease flowability in 3DCP with a constant w/b ratio, compared to PC-exclusive binder mixtures [46,58]. Moreover, 2% SF improved buildability and increased early strength, reinforcing that SF has a pozzolanic reaction at an early stage and accelerated cement hydration [58,111].

**Table 2:** Physical properties of different types of standard SCM used in 3DPCC mixtures.

Line Number	Type of SCM	Average Particle Size (µm)	Density (kg/m^3^)	Specific Surface Area (m^2^/g)	Ref.
1	Fly ash (Class C)	1.00	2620	-	[118]
2	Fly ash (Class F)	-	2600	-	[30]
3	Fly ash (Class F)	-	2600	-	[59]
4	Metakaolin	5.12	-	-	[53]
5	Metakaolin	11.69	2490	-	[54]
6	Calcined clay	69.35	2510	10.06	[55]
7	High-grade calcined clay (95%Mk)	3.75	2134	12.60	[56]
8	Low-grade calcined clay (50% MK)	69.35	2512	10.06	[56]
9	Low-grade calcined clay	69.35	-	-	[44]
10	Limestone filler	27.71	2710	-	[54]
11	Limestone powder	24.19	2650	1.22	[55]
12	Limestone powder	24.19	2646	1.22	[56]
13	Limestone powder	24.19	-	-	[44]
14	Silica Fume	-	2200	-	[30]
15	Silica Fume	0.10	2250	-	[118]
16	Silica fume	-	2200		[57]
17	Silica Fume	≤ 1.0	-	15–30	[46]
18	Ground granulated blast furnace slag	35	2990	-	[118]
19	Ground granulated blast furnace slag	-	2860	-	[59]

**Table 3:** Chemical composition of standard SCM used in 3DPCC mixtures.

Line Number	Material	SiO_2_	CaO	Al_2_O_3_	Fe_2_O_3_	SO_3_	MgO	Na_2_O	K_2_O	TiO_2_	ZrO_2_	CO_2_	P2O5	SrO	Other	LOI	Ref.
1	Fly ash (Class C)	58.64	2.78	25.94	6.55	0.40	1.16	0.24	2.46	-	-	-	-	-	-	-	[118]
2	Fly ash (Class F)	59.32	1.28	29.95	4.32	-	0.61	0.16	-	-	-	-	-	-	-	-	[30]
3	Fly ash	49.00	14.26	18.41	5.12	0.25	1.73	2.01	3.39	0.89	-	2.63	1.44	0.20	0.67	-	[52]
4	Fly ash (Class F)	51.10	5.80	28.8	6.40	1.00	1.10	0.30	1.20	2.50	-	-	-	-	1.20	-	[90]
5	Fly ash (Class F)	59.32	1.28	29.95	4.32	-	0.61	0.16	-	-	-	-	-	-	-	0.80	[59]
6	Metakaolin	58.18	3.89	23.33	0.68	0.08	0.84	9.78	-	-	-	-	-	-	-	1.90	[109]
7	Metakaolin	52.80	0.43	42.00	2.50	-	-	-	0.28	2.04	-	-	-	-	-	0.94	[53]
8	Calcined Clay	55.10	0.60	38.40	2.60	-	-	-	0.20	1.10	0.10	-	-	-	1.90	-	[55]
9	Low-grade calcined clay	55.10	0.60	38.40	2.60	-	-	-	0.20	1.10	0.10	-	-	-	1.90	-	[56]
10	Low-grade calcined clay	55.10	0.60	38.40	2.60	-	-	-	0.20	1.10	0.10	-	-	-	1.90	-	[44]
11	High-grade calcined clay	47.10	-	50.60	0.50	-	-	-	0.20	1.30		-	-	-	0.10	-	[56]
12	Limestone powder	0.20	39.6	-	0.10	-	-	-	-	-	-	-	-	-	60.10	-	[55]
13	Limestone powder	0.20	39.6	-	0.10	-	-	-	-	-	-	-	-	-	60.10	-	[56]
14	Limestone powder	0.20	39.6	-	0.10	-	-	-	-	-	-	-	-	-	60.10	-	[44]
15	Silica Fume	93.96	0.69	0.24	0.26	0.07	0.48	0.51	-	-	-	-	-	-	-	3.20	[109]
16	Silica Fume	96.80	0.40	-	-	-	-	-	-	-	-	-	-	-	-	-	[30]
17	Silica Fume	95.75	0.17	0.35	0.21	0.42	0.09	0.51	0.16	-	-	-	-	-	-	-	[118]
18	Silica Fume	95.01	0.35	0.82	1.86	0.32	1.24	-	-	-	-	-	-	-	-	1.58	[58]
19	GGBS	30.80	47.50	11.45	2.26	3.03	3.65	0.27	-	-	-	-	-	-	-	2.60	[109]
20	GGBS	35.78	43.86	12.31	1.02	2.71	9.33	0.24	0.42	-	-	-	-	-	-	-	[118]
21	GGBS	30.95	27.95	24.20	0.30	-	10.57	0.22	-	-	-	-	-	-	-	3.87	[59]
22	GGBS	29.65	39.37	15.56	0.35	4.32	7.54	0.45	0.51	-	-	-	-	-	-	0.36	[47]
23	GGBS	31.86	39.55	15.97	0.23	1.14	9.27	0.41	0.31	0.64	-	-	-	-	-	1.23	[119]

GGBFS has also shown to be an option for designing 3DPCC [47,59,112,119,120]. GGBFS is a by-product resulting from the manufacturing process of pig iron at temperatures between 1170 and 1500 °C [115]. This waste is predominantly silica (29.65–35.78% wt.) and calcium oxide (27.95–47.50% wt.), as can be seen in Table 3 (lines 19 to 23). Panda et al. [47] studied 3DPCC with a high volume of GGBFS (70–84% wt. binder) and observed that the increase in slag decreases yield stress. A 3DPCC with the lowest GGBFS (70%) and higher amounts of PC (20% wt.) and hydrated lime powder (10% wt.) showed a substantial increase in yield stress with a reduction in GGBFS [47]. 

#### 5.2.2. Unconventional SCM

Unconventional SCM are mainly waste, or by-products materials intended for landfills and have no added value. In this way, their incorporation into 3DPCC is an opportunity to keep these non-valuable materials in the value chain, creating new industrial synergies. Unconventional SCM has also been studied in 3DPCC, but the research level is still very limited compared to those studied in standard SCM. 

Table 4 and Table 5 summarise the main physical and chemical properties of unconventional SCM used in 3DPCC. It follows some discussion here. RHA with an average particle size between 2 and 3 µm after griding (Table 4, line 1) and mainly composed of silica (89.89% wt.) has been studied to produce 3DPCC, as presented in Table 5 (line 1). RHA could replace 20% wt. of PC with positive effects on rheology and printability but required higher water content and superplasticizer dosage [61]. In addition, 20% of RHA provides a substantial increase in static yield stress up to 30 min after mixing, showing a filler effect and pozzolanic reaction in cement hydration. RHA also improved buildability compared to exclusive binder PC mixtures [61]. However, RHA is not available worldwide.

Fly and bottom ashes from incineration of MSW as PC replacement (5, 7.5, 10 and 15% wt. binder) also showed reasonable results in 3DPCC [60]. Replacement of PC with MSW ashes decreased the workability and setting time. This decrease was more significant for fly ash due to its finer particles (average particle size 29.2 µm) compared to bottom ashes (average particle size 732 µm), and the presence of alite in crystalline phases. Fly ash from MSW incineration increased yield stress in 3DPCC and poor layer stability [60]. At early ages (before 7 days), MSW fly ash incorporation up to 10% wt. increased compressive strength compared to the reference 3DPCC with only PC as binder, due to the presence of amorphous silica in the XRD pattern, providing pozzolanic activity, and finer particles responsible for the filling effect. Fly ash can be beneficial for shape stability, and the construction rate of 3DPCC increased [60]. On the other hand, bottom ashes seem more viable to be used as an aggregate due to the higher particle size (see Table 4, lines 2 and 3).

Recycled powder from CDW with a particle size <160 µm (Table 4, lines 4 and 5) and with high quantities of silica (25.62–45.71% wt.) and calcium oxide (21.99–41.22% wt.) (Table 5, lines 4 and 5) was also studied as an unconventional SCM. The recycled powder from CDW was used as a cement replacement in 10, 20 and 30% wt. and slightly decreased flowability [62,63]. Zhang et al. [62] determined a flowability reduction of 2.3% to 4.4% with 10 and 20% wt. recycled powder, respectively, compared to control mixtures. This unconventional SCM decreased the open time and initial and final setting time in 3DPCC [63]. Open time for mixtures with 10, 20 and 30% wt. recycled powder was 40, 25 and 15 min, respectively. The initial and final setting time of 3DPCC without recycled powder was up to 6 and 8 h, and the initial and final setting time of a mixture with 30% wt. recycled powder was 3.2 and 4.6 h, respectively [63]. In terms of hardened properties, 10 and 20% of recycled powder from CDW as PC replacement decreased compressive strength (5.4% and 14.8%, respectively) and flexural strength (4.4 and 13.6%, respectively) and positively reduced drying shrinkage at 60 days (3.3 and 12.4%, respectively) [62]. In addition, Qian et al. [64] compared GGBFS and recycled powder (particle size inferior to 0.16mm) as SCM in 3DPCC with recycled fine aggregate. Replacement of GGBFS with recycled powder from CDW at 0 and 10% wt. increased yield stress (from 1423 Pa to 1573 Pa, respectively) and viscosity remains, while 20% wt. of replacement decreased yield stress to 1173 Pa. On the other hand, recycled powder from CDW as GGBFS replacement decreased the compressive strength of 3DCP [64]. 

### 5.3. Aggregates

The construction industry has a high demand for aggregates; therefore, alternative materials should be regarded as decreasing excessive consumption of this non-renewable resource. Secondary aggregates from by-products and waste can be an option. However, their use may be invalidated due to the environmental impact of transport distance and the required mechanical and chemical treatments. 

The aggregates generally adopted in 3DCP are mainly fine silicious or quartz sand, with a maximum particle size of 2 mm, as mentioned in Section 4. Generally, finer aggregates increase yield stress and buildability and decrease extrudability in mixture designs with constant aggregate volume using printing equipment with a screw extruder [89]. Further, if the aggregate volume fraction increases, an increase in required pumping pressures is needed [121] due to the increase in viscosity and loss of flowability. However, there is still a gap in studying the effect of aggregate fraction in 3DPCC, such as volume, particle size and origin. 

Even though 3DPCC is usually a mortar scale, some efforts have been made to use coarse aggregates [122]. One of the main challenges is that the pumpability and extrudability decrease. Moreover, 3DPCC with coarse aggregates may have lower compressive strength [123,124]. In this field, extensive experimental research is required to ensure the printability of coarse aggregate in 3DPCC. Still, most pumping and printhead machines used for 3DPCC are not applicable if the coarse aggregate is employed and new developments are necessary [4]. 

Recycled aggregates from CDW have been under scrutiny as an aggregate replacement in 3DPCC. The construction industry generates enormous quantities of waste (37% of total waste generated by households and business activities in EU-27 in 2020) [125], making CDW available in large volumes in a wide geographical dispersion. For 3DCP applications, fine aggregates from CDW are particularly interesting. Recent research has emerged which focusses on different particle sizes of recycled aggregates from CDW, from 0.9 to 12mm, as summarized in Table 6. Replacing river sand with recycled sand from CDW (d < 0.9 mm) decreased extrudability and flowability of 3DPCC [77,78]. Flowability loss was more evident for higher percentages of replacement [75], while percentages between 0 and 50% of recycled sand presented a lower reduction of flowability [84]. Flowability is affected by higher water absorption of recycled sand from CDW compared to river sand, between 12.1 and 15.0% (Table 6). However, flowability and extrudability of 3DPCC with recycled sand can be improved by increasing the superplasticiser dosage [76,77], adding sodium gluconate as a retarder [78,79], or implementing an agitator to create a secondary mixing process capable of reducing the consistency of concrete when it is subjected to disturbance [80]. In addition, recycled sand significantly increases the static yield stress and viscosity when replacing river sand at 50 and 100% [79]. Nonetheless, recycled sand as natural fine aggregate replacement decreased compressive strength [75,83,84,85]. Total replacement of natural aggregate can reduce 60.3% of compressive strength [83], but a replacement of 12.5% wt. decreased compressive strength by 1.4% [84], compared to reference mixture at 28 days. Similar results were determined for flexural strength [75,77,82,83]. Natural coarse aggregate replacement with recycled coarse aggregate from CDW (8–12mm) also decreased the flowability of 3DPCC [126,127]. Nevertheless, Xiao et al. [127] also noted that flowability loss with recycled coarse aggregate incorporation, due to the higher water absorption (see Table 6) and rough particle shape, may be reversed by adjusting the superplasticiser dosage [127]. In this regard, recycled coarse aggregate increases the yield stress of 3DPCC [126,128,129] and is more prominent for higher percentages [126,128]. Recycled coarse aggregate led to a compressive and flexural strength reduction and a slight increase in porosity [128]. In addition, the deposition process layer by layer causes an increase in porosity between the interlayer and intralayer interface, and recycled coarse aggregate increases the roughness of the filament surface of the 3D printed concrete, increasing the susceptibility of existing large-volume pores into the interlayer interface area [130].

Mining and quarrying industries generate significant amounts of solid waste with the potential to be used as a partial natural aggregate replacement in 3DPCC, such as mine tailing and OSW. Table 7 and Table 8 show the physical and chemical properties of these types of waste previously incorporated in 3DPCC, respectively. Some discussion follows on the effect of such wastes on 3DPCC when used as natural aggregate replacement. 

Mine tailing, with a mineralogically composition of silica (80%), calcite (15%), and feldspars (15%) (Table 8, lines 1 and 2), could be a feasible unconventional aggregate and fulfills 3DPCC requirements in terms of workability, consistency, buildability, and strength, when replacing natural aggregate up to 20% wt. [131]. 

Copper tailing, finer than sand (Table 7, lines 2 and 3 and Table 8, lines 1 and 2), can modify the granulometric curve of aggregates and maintain a satisfactory extrudability of 3DPCC without disruption, segregation and blockage printing [86]. Moreover, flowability increases when copper tailing replaces sand (from 0 to 50% by weight), more evident for 40 and 50% wt. (increasing 64 and 69%, respectively), with a stiffness decrease during the early ages. Lower percentages of copper tailing did not influence open time, 90 min (from 0 to 20% wt.), while higher percentages reduced open time to 70 min [86]. Ma et al. [86] determined that 30% wt. of copper tailing as natural sand replacement is optimal for buildability and mechanical strength. For several combinations of iron and copper tailing as aggregate, Li et al. [87] determined the optimal aggregate combination of 80% wt. iron tailing and 20% wt. copper tailing (corresponding to 50% total mixture) for a constant water-to-solid ratio. Flowability and initial and final setting time reduced when copper tailing content increased and iron tailing decreased because copper tailing had a finer particle size and larger water absorption. Furthermore, 3DPCC with iron and copper tailing did not show environmental issues of leaching toxicity and radioactivity [87]. 

OSW from quarrying and transformation activity seemed an option to be used as natural aggregate replacement in 3DPCC [132], and its potential has been widely proven in conventional cementitious materials, not only as an aggregate replacement [133,134,135,136,137], but also as SCM [138,139,140]. OSW from the cutting process is a promising alternative because mechanical treatment is not required, and particle size is smaller than 600 µm (Table 7, line 5). However, there are also difficulties in its incorporation due to the high moisture content and heterogeneity [132,141,142]. OSW as a river sand replacement in 3DPCC required a higher superplasticiser dosage to ensure workability [65], but replacement ratios of 5, 10 and 15% wt. provided favourable results for compressive and flexural strength [65].

Glass waste (GW) may be also a feasible unconventional aggregate for more sustainable 3DPCC [66,67,68,69,143]. Sand replacement with GW (particle size < 2 mm) at 25, 50, 75 and 100% wt. increased dynamic yield stress and plastic viscosity and decreased static yield stress for 3DPCC with a w/b ratio of 0.35 and aggregate-to-binder ratio of 0.8 [66]. The total replacement of sand with GW increased 18.2% the dynamic yield stress of 3DPCC, compared to 3DPCC without GW. Even though the GW incorporation reduced pumpability, mixtures with 100% GW as aggregate still showed satisfactory pumpability through the 3D printing system. Nonetheless, static yield stress was slightly reduced because of inter-particle friction reduction caused by the smooth surface of the GW [66]. 

Cuevas et al. 2021 [67] concluded that the total replacement of basalt aggregate with GW in 3DPCC also increased yield stress and viscosity (3.5% and 58.9%, respectively) and reduced the initial and final setting time due to filler and pozzolanic effect. However, there is a need to decrease SCM (limestone filler) content and increase water content to find a proper flowability in 3DPCC with GW (50 and 100% by volume) due to the finer particles which result from the milling processes and higher water absorption [67]. 

Unconventional aggregates from rubber waste have also been incorporated as a natural aggregate replacement in 3DPCC [70,71,72]. Ye et al. [70] determined the optimum percentage of 40% vol. of crumb rubber (particle size < 125 µm) as a silica sand replacement for ultra-high ductile 3DPCC. Crumb rubber required a higher dosage of water reducer to find adequate extrudability and buildability when incorporation dosage increases at a constant w/b ratio [70]. Rubber powder and rubber granules have hydrophobic characteristics and a larger particle size than limestone sand, decreasing water needs by 23% in 3DPCC with rubber powder (25% vol.) and rubber granules (75% vol.) when totally replacing limestone sand [71]. Therefore, adjusting water content in the function of printability was needed to control fluidity and achieve adequate rheology [71]. Mechanical properties and bulk density are significantly affected by rubber incorporation [71]. In addition, recycled Polyethylene Terephthalate (PET), a type of thermoplastic polymer, was also incorporated as aggregate in 3DPCC [73], demonstrating that 10% vol. recycled PET does not significantly affect compressive strength at early ages. However, higher percentages of recycled PET (30 and 50% vol.) decreased compressive strength at early ages and 28 days.

From steel manufacturing, unconventional aggregates such as steel slag can also be an option in 3DPCC [84]. Natural sand replaced with 25% wt. steel slag decreases fluidity and increases friction between particles due to its irregular shape, roughness, and more porous surface [74]. Steel slag had a good effect on the interfacial bonding behaviour of 3DPCC and resulted in a compacted structure. It improved compressive strength at 7 and 28 days. Dai et al. [74] also showed that weaker mechanical results at 3 and 7 days could be improved by adding sodium sulfate (0.5 and 1.0%) [74].

### 5.4. Admixtures

The emergence of superplasticisers was a breakthrough for concrete technology, allowing the efficient dispersal of cementitious particles while keeping a very low water content. This gave rise to advanced cement-based composites, such as self-compacting concrete [144], UHPC [145] and, more recently, 3DPCC.

Generally, the water-to-binder weight ratio is relatively low in 3DPCC (w/b = 0.37 on average, from the literature analysis presented in Section 4). Therefore, superplasticiser is needed to ensure the pumpability and extrudability while maintaining a high mechanical strength, maintaining the required consistency for each material process. 

Several types of superplasticisers are commercially available, with different molecular structures and, consequently, different effects on the rheology of fresh 3DPCC. The superplasticizer dosage and type have a key role in rheological behavior and the robustness of such rheological properties and, consequently, influences its compatibility with different cement and SCM [146]. For example, naphthalene sulfonate formaldehyde polycondensate superplasticiser has a linear structure and has the ability to decrease the attraction of particles through an electrostatic repulsion; on the other hand, polycarboxylic ether superplasticiser has a comb-like structure and decreases the attraction of particles through steric hindrance [146,147]. Viscosity Enhancement Agent (VEA) is also commonly incorporated to improve the fluidity and cohesion of fresh cement-based materials [148]. Similar to superplasticiser, the effectiveness of VEA in terms of rheological properties also substantially depends on its type [146,147]. 

The buildability of 3DPCC can be enhanced by incorporating setting and hydration accelerators which increase the static yield stress of printable mixtures [149,150]. However, accelerators may present collateral effects depending on the type of accelerator used. Incorporating accelerators in cement-based mixtures also affected the interlayer bond strength and, consequently, the hardened state properties and durability [151]. When any accelerator is used in 3DPCC an adjustment of superplasticiser dosage is required through iterative experimental methods to guarantee good printability and a satisfactory open time. However, the negative effect of incorporating accelerators is linked to the loss of pumpability and extrudability in the case of a longer printing process.

Alternative admixtures and additions have also been studied in 3DPCC to accelerate the restructuration kinetics and increase buildability, such as attapulgite nano clay and nano-silica [112]. Although these additions also affected extrudability, particularly after the interruption of the printing process, giving material sufficient time for structural build-up, this effect can be reversible with external shear or vibration. 

Other admixtures with a multi-purpose, such as cellulose ethers, are also used in 3DPCC for viscosity control of the mixture to improve water retention in 3DPCC layers elements and improve bond strength and buildability [152]. Retarders are also incorporated in 3DPCC to extend the open time window during printing. For example, sucrose, a usual retarder, can retard cement hydration [151], decrease the yield stress and viscosity, and extend the limits of pumping and extrusion time.

## 6. Conclusions and Future Needs

The emerging technology, 3DCP, has shown promising results for improving productivity and overcoming the current limitations of the construction industry. However, there are still several issues which require attention. At the material level, one of the main challenges is the design of sustainable and circular printable cement-based composites. Indeed, as discussed in Section 3, the environmental impact of 3DPCC is highly dependent on the cement dosage required to ensure printing ability parameters compared to conventional cast concrete. 

Although decreasing the amount of cement may negatively affect 3DPCC buildability, this challenge has been circumvented by replacing cement with SCM, providing binary, ternary and quaternary blends. For a total of 157 mixture designs available in the literature survey, the mean and median values of cement-to-binder weight ratio are 0.77 and 0.93, respectively, highlighting opportunities of using standard and unconventional SCM as a partial cement replacement to fulfil the fine powder need of 3DPCC. Most studies have employed standard SCM, based on local availability, mainly FA, SF and LF. The use of unconventional SCM is still limited, but it shows that a new and feasible tendency is emerging. Unconventional SCM, such as RHA, fly ash from MSW incineration and recycled powder from CDW, were used to replace cement between 0 and 30% wt. as an alternative to standard SCM. SCM allows an even more significant reduction in cement quantity and has advantages in the environmental impact of 3DCP. Therefore, using locally available SCM from waste or by-products is a key opportunity for saving energy, reducing cost, and decreasing CO_2_ emissions associated with 3DPCC production, particularly the valorization of local wastes. 

The aggregate used in 3DPCC mixture designs is often limited to a particle size of 2mm because of the constraints of the printer equipment. Moreover, fine aggregate requires intensive mechanical energy to create finer granulometric fractions. For this reason, it is important to find alternative materials to replace also conventional aggregates. Waste and by-products, such as OWS, mine tailing, glass waste, crumb rubber, steel slag, recycled sand and CDW, seemed to be feasible options. 

Because 3DPCC can extend the value chain of wastes and by-products, it can also lead to interesting industrial synergies. Nevertheless, these upcycling strategies require more LCA analysis to verify sustainability in line with Circular Economy principles. LCA cementitious structural or non-structural elements manufactured by 3D printing are crucial to understanding the real environmental impact of 3DPCC and final elements compared with conventional concrete elements. In addition, when materials from waste and by-products are evaluated, LCA has to include mechanical and chemical treatments, if necessary, and transport distance. 

## Figures and Tables

**Table 1:** Chemical, physical, and mechanical requirements for various SCM used in concrete.

	EN 13263-1	EN 450-1			EN 151671-1	NP 4220	ASTM C 618		
	Silica Fume	Coal Fly Ash			Slag	Pozzolans in General	Fly Ash and Pozzolan		
		A	B	C			N	F	C
SiO_2_ (%)	>85	>25			-	-			
SiO_2_ + Al_2_O_3_ + Fe_2_O_3_ (%)		>70			-	-	>70	>70	>50
Free CaO (%)		≤2.5			-	-	-	-	-
Reactive CaO (%)		≤10			-	-	-	-	-
CaO (%)	<1.0		-			<10.0	-	-	-
CaO + MgO + SiO_2_ (%)			-		>66.6	-	-	-	-
(CaO + MgO)/SiO_2_ (%)			-		>1	-	-	-	-
MgO (%)		≤4.0			≤18	-	-	-	-
Cl (%)	<0.3	<0.1			<0.1	<0.1	-	-	-
SO_3_ (%)	≤2.0	≤3.0			<2.5	<3.0	<4.0	<5.0	<5.0
(Na_2_O)_eq_ (%)		≤5.0			-	<5.0	-	-	-
Loss on Ignition (%)	≤4.0	≤5.0	≤7.0	≤9.0	<3.0	≤5.0	<10	<6.0	<6.0
Organic matter (%)			-		<0.2	-	-	-	-
Sulphide (%)			-		<2.0	-	-	-	-

**Table 4:** Physical properties of different types of waste used in 3DPCC mixtures as unconventional SCM.

Line Number	Material	Maximum Particle Size (mm)	Average Particle Size (µm)	Specific Gravity	Ref.
1	Rice husk ash	-	2–3	-	[61]
2	Fly ash from MSW incineration	-	29.2	1.89	[60]
3	Bottom ash from MSW incineration	-	732	1.76	[60]
4	Recycled powder from CDW	150	-	-	[62]
5	Recycled powder from CDW	160	-	-	[64]

**Table 5:** Chemical composition of different types of waste or by-products used as unconventional SCM used in 3DPCC mixtures.

Line Number	Material	SiO_2_	CaO	Al_2_O_3_	Fe_2_O_3_	SO_3_	MgO	Cl	Na_2_O	K_2_O	TiO_2_	CO_2_	Others	LOI	Ref.
1	Rice husk ash	89.89	1.05	0.11	0.28	0.40	0.13	-	-	2.92		3.84	-		[61]
2	Recycled powder from CDW	38.61	41.22	7.13	3.19	1.04	1.35	0.04	-	-	-	-	7.42	-	[62]
3	Recycled powder from CDW	45.71	21.99	15.83	6.77	2.29	2.71	-	1.14	2.56	0.85	-	-	-	[63]
4	Recycled powder from CDW	25.62	30.81	5.27	2.02	1.35	2.15	-	0.413	1.04	0.228	-	-	30.71	[64]

**Table 6:** Physical properties of different recycled aggregates from CDW used in 3DPCC mixtures.

Type of Waste	Maximum Particle Size (mm)	Minimum Particle Size (mm)	Fineness Modulus	Apparent Density (kg/m^3^)	Loose/Dense Packing Density (kg/m^3^)	Moisture Content (%)	Water Absorption (%)	Crushning Value (%)	Specific Gravity	Ref.
Recycled sand	0.90	-	1.53	2411	1014/1070	0.6	13.5	-	-	[75]
Recycled sand	0.90	-	-	-	-	-	15.0	-	-	[76]
Recycled Sand	0.90	-	1.53	2411	1014/1070	0.6	15.0	-	-	[77]
Recycled sand	0.90	-	1.53	2411	1014/1070	0.6	13.5	-	-	[78]
Recycled fine aggregate	0.90	-	1.53	2411	1014/1070	0.6	13.5	-	-	[79]
Recycled fine aggregate	0.90	-	1.53	2411	1014/1070	0.6	13.5	-	-	[80]
Recycled fine aggregate	0.90	-	-	-	-	-	13.5	-	-	[81]
Recycled sand	2.36	-	2.16	2411	1014/1070	0.6	13.5	-	-	[82]
Recycled sand	2.36	-	2.16	2411	1014/1070	0.6	13.5	-	-	[62]
Recycled fine aggregate	2.00	-	-	-	-	-	12.1	-	-	[83]
Recycled fine aggregate	-	-	-	2381	-	0.5	13.7	-	-	[84]
Recycled fine aggregate	4.75	0.15	3.50	2272	-	7.9	13.3	-	-	[127]
Recycled brick aggregate	4.75	-	-	2708	-	-	18.2	-	-	[85]
Recycled coarse aggregate	8.00	-	5.60	-	1372/-	-	4.4	29.4	2.39	[129]
Recycled coarse aggregate	-	-	-	-	-	-	7.3	23.3	-	[126]
Recycled coarse aggregate	10.00	4.75	-	2634	-	4.0	7.9	18.7	-	[127]
Recycled Coarse aggregate	12.00	5.00	-	2538	-	-	7.3	23.3	-	[128]
Recycled Coarse aggregate	12.00	5.00	-	-	-	-	7.3	23.3	-	[130]

**Table 7:** Physical properties of different types of waste used as aggregate in 3DPCC mixtures, excluding recycled aggregates.

Line Number	Type of Waste	Maximum Particle Size (µm)	D_50_ (µm)	Fineness Modulus	Specific Surface Area (m^2^/g)	Specific Gravity	Ref.
1	Mine tailing	250	-	-	-	-	[131]
2	Copper tailing	-	123.75	-	0.141	-	[86]
3	Copper tailing	-	33.13	-	-	-	[87]
4	Iron tailing	-	75.65	-	-	-	[87]
5	OSW	600	12.67	1.15	-	2.61	[65]
6	Recycled glass	1700	-	-	-	-	[69]
7	Recycled glass	1000	-	-	-	2.53	[67]
8	Crumb rubber	125	-	-	-	-	[70]
9	Rubber powder	1000	-	-	-	1.209	[71]
10	Rubber granules	4000	-	-	-	1.195	[71]
11	Recycled PET bottles	-	-	-	-	1.4	[73]
12	Steel slag	-	-	1.01	-	-	[74]

**Table 8:** Chemical composition of different types of waste used as aggregate in 3DPCC mixtures, excluding recycled aggregates.

Line Number	Material	SiO_2_	CaO	Al_2_O_3_	Fe_2_O_3_	SO_3_	MgO	Na_2_O	K_2_O	TiO_2_	P2O5	SrO	MnO	BaO	CuO	Cr_2_O_3_	ZnO	PbO	LOI	Ref.
1	Copper tailing	39.77	22.29	4.61	20.16	3.05	7.17	1.32	0.44	-	0.26	-	0.23	-	-	-	-	-	-	[86]
2	Copper tailing	47.92	14.14	14.44	6.00	1.07	1.44	2.04	3.38	1.32	0.51	-	-	0.54	0.08	0.01	0.01	0.01	-	[87]
3	Iron tailing	42.06	10.50	11.51	15.50	-	2.54	0.58	5.37	0.59	0.17	-	-	0.06	0.06	0.00	0.01	-	-	[87]
4	OSW	21.06	49.23	3.64	15.64	-	3.65	-	3.03	1.54	-	1.94	0.25	-	-	-	-	-	-	[65]
5	Steel Slag	17.18	40.21	3.05	21.72	-	7.22	-	-	0.91	2.77	-	3.14	-	-	-	-	-	2.81	[74]

## Data Availability

All related data is part of the manuscript.

## Abbreviations

3DCP	3D Cementitious materials Printing
3DPCC	3D Printable cement-based composites
AM	Additive manufacture
CSH	Calcium Silicate Hydrates
CDW	Construction and Demolition Waste
FA	Fly Ash
GDP	Gross Domestic Product
GGBFS	Ground Granulated Blast Furnace Slag
GHG	Greenhouse Gases
h	hours
HEMC	Hydroxyethylmethyl Cellulose
HPMC	Hydroxy Propyl Methyl Cellulose
HR	Relative Humidity (%)
LC2	Limestone-calcined clay
LF	Limestone filler
LOI	Loss on ignition (%)
MSW	Municipal Solid Waste
OVC	Ordinary vibrated concrete
OSW	Ornamental Stone Waste
PC	Portland cement
RHA	Rice Husk Ash
SAI	Strength activity index (%)
SCM	Supplementary cementitious materials
SDG	Sustainable Development Goals
SEM	Scanning electron microscopy
SF	Silica Fume
SHCC	Strain-Hardening Cementitious Composites
UHPC	Ultra high-performance concrete
VMA	Viscosity Modifying admixture
w/c	Water-to-cement weight ratio
w/b	Water-to-binder weight ratio
